# Native Kidney Hydronephrosis Is Associated with Upper Urinary Tract Urothelial Carcinoma in Post-Kidney Transplantation Patients

**DOI:** 10.3390/jcm10194474

**Published:** 2021-09-28

**Authors:** Cheng-Ju Ho, Yu-Hui Huang, Tzuo-Yi Hsieh, Min-Hsin Yang, Shao-Chuan Wang, Wen-Jung Chen, Tsung-Hsien Lee, Wen-Wei Sung, Sung-Lang Chen

**Affiliations:** 1 Department of Urology, Chung Shan Medical University Hospital, Taichung 402, Taiwan; benaries108@hotmail.com (C.-J.H.); joe.hsieh46@gmail.com (T.-Y.H.); barbarian06070136@gmail.com (M.-H.Y.); rosenbeck.wang@gmail.com (S.-C.W.); mimic1024@yahoo.com.tw (W.-J.C.); flutewayne@gmail.com (W.-W.S.); 2 Institute of Medicine, Chung Shan Medical University, Taichung 402, Taiwan; jackth.lee@gmail.com; 3 Department of Physical Medicine and Rehabilitation, Chung Shan Medical University Hospital, Taichung 402, Taiwan; yhhuang59@hotmail.com; 4 School of Medicine, Chung Shan Medical University, Taichung 402, Taiwan; 5 Department of Obstetrics and Gynecology, Chung Shan Medical University Hospital, Taichung 402, Taiwan

**Keywords:** kidney transplantation, hydronephrosis, upper urinary tract, urothelial carcinoma

## Abstract

Background: Upper urinary tract urothelial carcinoma (UTUC) is the most common malignancy occurring after kidney transplantation (KT) in Taiwan. The aim of this study was to investigate the association between native kidney hydronephrosis and UTUC in post-KT patients. Methods: From 2003 to 2018, we conducted a retrospective cohort study that enrolled 1005 post-KT patients, 67 of whom were subsequently diagnosed with UTUC. We divided patients into two groups based on whether or not they had UTUC. Multivariate analysis and Kaplan-Meier plot were used to evaluate if native kidney hydronephrosis was associated with post-KT UTUC. Results: The total cohort consisted of 612 men (60.9%) and 393 women (39.1%) with a mean age of 48.2 ± 12.0 at KT. The mean follow-up time was 118.6 ± 70.2 months, and mean time from KT to UTUC was 7.53 years. There was a significant gender difference with a female predominance among the UTUC patients (73.1% versus 26.9%, *p* < 0.001). Native kidney hydronephrosis occurred more frequently in the UTUC group (68.7% versus 4.8%, *p* < 0.001). Multivariate analysis showed that native kidney hydronephrosis and female gender were significantly associated with UTUC with odds ratios of 35.32 (95% CI, 17.99–69.36; *p* < 0.001) and 3.37 (95% CI, 1.55–7.29; *p* = 0.002), respectively. UTUC in the post-KT patients also showed aggressive pathological characteristics and a tendency toward bilateral lesions (41.8%). Conclusions: Native kidney hydronephrosis is significantly associated with post-KT UTUC patients in Taiwan. Native kidney hydronephrosis may be a deciding factor for standard nephroureterectomy and bladder cuff excision in selected patients. Nevertheless, almost half of the patients with kidney hydronephrosis do not present with UTUC at the end of our study.

## 1. Introduction

For end-stage renal disease (ESRD) patients, kidney transplantation (KT) is an important treatment in renal replacement therapy. With the benefits from advances in medical procedures and immunosuppressive agent use, long-term overall survival and graft survival in KT recipients have improved. However, KT recipients are known to have a higher incidence of de novo malignancy as compared with the general population because of their use of immunosuppressive agents and chronic inflammatory status. It remains one of the lingering causes of mortality among KT recipients [1].

Among different ethnic populations and geographic regions, the types of post-KT malignancy vary. Skin cancer and lymphoproliferative disorder are frequent post-KT cancers in Western countries. In contrast, high incidence (3.5~4.1%) of urothelial carcinoma (UC) in Taiwan has been elucidated before [1,2,3,4,5]. The incidence of upper urinary tract urothelial carcinoma (UTUC), ranking as the most common form of post-KT malignancy in Taiwan, is higher than that of urinary bladder urothelial carcinoma (UBUC) [2,3,4,5]. Possible risk factors are the use of Chinese herbs and arsenic in groundwater, and these cases may represent over one half of the post-KT malignancies found in previous studies [2,4].

Thus, the early detection of UTUC in post-KT patients is an important issue for improving oncologic outcomes. However, hematuria as a diagnostic basis for UTUC in post-KT patients has shown disappointing results. To make matters worse, it often delays diagnosis [6]. Nevertheless, native kidney parenchyma atrophy and native ureter stricture in post-KT recipients usually preclude diagnostic ureteroscopy (URS) and simultaneous tissue proof. Periodical renal sonography follow-up is routine for post-KT patients. Kidney hydronephrosis had been reported as a risk factor for developing UTUC in ESRD patients [7,8,9]. The role of kidney hydronephrosis in post-KT patients with UTUC has yet to be well clarified. Therefore, we conducted a retrospective cohort study to compare post-KT patients with and without native kidney hydronephrosis and their subsequent risk for UTUC. We also investigated the clinical parameters that were correlated with UTUC after KT.

## 2. Materials and Methods

This retrospective cohort study was approved by the Institutional Review Board of Chung Shan Medical University Hospital (IRB number: CS1-20219). We enrolled cases of patients over 18 years of age with regular medical visit records or lost follow-up for a known reason, and a systemic examination was carried out to confirm that no malignancy, including UC, had existed before KT. From January 2003 to September 2018, we retrospectively analyzed 1005 KT patients in our hospital, 67 of whom were subsequently diagnosed with UTUC. The immunosuppressive agent protocol consisted of a calcineurin inhibitor (cyclosporine or tacrolimus), mycophenolate mofetil or azathioprine, and steroids without CD3 monoclonal antibody or anti-thymocyte globulin induction therapy. The kidney transplantation follow-up protocol at our institution included blood examination, routine urine and sediment analysis, and urine cytology monthly for 6 months and then quarterly thereafter. Post-KT renal sonography was performed every 3 months during the first year, then biannually. Hydronephrosis in a native or graft kidney was defined based on an image interpretation or documented image report in the medical records. Patients with kidney hydronephrosis before KT were excluded. Unilateral or bilateral native kidney hydronephrosis was generally categorized to the hydronephrosis group. In patients with hematuria or kidney hydronephrosis in native kidneys, we performed cystoscopy with bilateral retrograde pyelography or URS. Urine cytology was collected with voiding or washing during URS. Abdominal computed tomography (CT) scan was performed when the findings from the above-mentioned examinations were inconclusive or abnormal. Smoking and hypertension histories were obtained from the medical record documentation. Elderly was defined as age >65 years. Other patient characteristics, such as diabetes, dyslipidemia, hyperuricemia, hepatitis B virus (HBV), hepatitis C virus (HCV), and BK virus infection diagnosis, were based on laboratory examinations or serological tests. A UTUC diagnosis was confirmed by a peer reviewed pathological examination, and specimens were obtained by initial URS biopsy or subsequent nephroureterectomy. Once patients had been proved to have UTUC by URS biopsy or suggestive UTUC by image study, simultaneous bilateral nephroureterectomy and bladder cuff excision were the standard surgical procedures in our cohort.

Continuous data were analyzed using *t* tests, and chi-square tests were used to compare the categorical variables in the different groups. Univariate analysis was conducted and significant risk factors from univariate analysis were put in multivariate analysis. Multivariate logistic regression analysis was used to determine the factors significantly associated with UTUC. Kaplan-Meier curves and log-rank tests were used to compare the time to UTUC events between the post-KT cohorts with and without native kidney hydronephrosis. Statistical analysis was performed using SPSS (SPSS for Windows Version 16.0, SPSS Inc., Chicago, IL, USA). A two-tailed *p*-value of <0.05 was regarded as statistically significant.

## 3. Results

The total cohort consisted of 1005 KT patients, including 612 men (60.9%) and 393 women (39.1%), with a mean age of 48.2 ± 12.0 years old at KT (Table 1). The mean length of follow-up was 118.6 ± 70.2 months with 47.1% of patients with >10 years of follow up. At the end of the study, 82 patients had graft failure and were receiving hemodialysis (5 patients in the UTUC group and 77 patients in the non-UTUC group). Common systemic diseases including hypertension, diabetes mellitus, dyslipidemia, and hyperuricemia all had a prevalence above 50%. For viral infections, HBV and HCV infections were observed in approximately 31.3% and 19.1% of patients, respectively, and the BK virus infection rate was around 39%. Hydronephrosis rates were 9.4% and 23.1% in native and graft kidneys, respectively. There were 84.1% (74/88) unilateral native kidney hydronephrosis cases and 15.9% bilateral native kidney hydronephrosis cases in our cohort.

Table 2 shows the basic characteristics of the KT patients with and without UTUC. A subsequent 67 patients were diagnosed with UTUC with proved pathological specimens during the follow-up period. The age of the patients in the UTUC group when they had undergone KT was older than that of the non-UTUC group (51.03 ± 9.58 versus 47.99 ± 12.08, *p* = 0.046). The mean age of UTUC diagnosis was 57.6 ± 11.3. The mean length from KT to UTUC was 7.35 ± 4.72 years. There were more females in the UTUC group (73.1% females versus 36.7% males, *p* < 0.001). Smoking histories were obtained via electronic medical records and showed lower rates in the UTUC group (11.3% versus 23.8%, *p* = 0.025). Dyslipidemia was commonly noted in the post-KT patients but was more prominent in the UTUC group (95.5% versus 81.0%, *p* = 0.003). However, there were no significant differences for hypertension, diabetes mellitus, hyperuricemia, HBV infection, HCV infection, or BK virus infection between the two groups. There were 46 cases with native kidney hydronephrosis, 39 unilateral (84.8%) and 7 bilateral (15.2%), among the 67 patients in the UTUC group. Native kidney hydronephrosis occurred more frequently in the UTUC group than the non-UTUC group (68.7% versus 4.8%, respectively, *p* < 0.001). However, graft kidney hydronephrosis was not significantly different between groups (20.9% versus 23.3%, respectively, *p* = 0.652) (Table 2).

**Figure 1:**
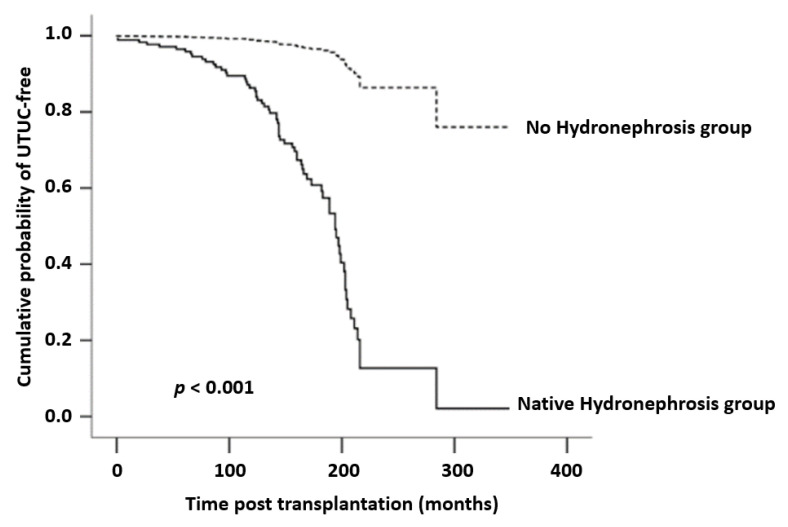
Kaplan-Meier analysis of cumulative probability of UTUC-free between with or without native kidney hydronephrosis groups. UTUC-free is statistically significant higher in the group of without native kidney hydronephrosis.

We conducted a multivariate logistic regression analysis for the significant risk factors from univariate analysis. As compared with the reference cohort, the group of post-KT patients with native kidney hydronephrosis had a higher overall risk for UTUC (OR, 35.32; 95% CI, 17.99–69.36; *p* < 0.001), and women also showed a statistically higher risk for the development of post-KT UTUC (OR, 3.37; 95% CI, 1.55–7.29; *p* = 0.002) (Table 3). Figure 1 demonstrates the comparative trend of native kidney hydronephrosis for UTUC using a Kaplan-Meier graph and details of log rank test *p* value < 0.001.

We also demonstrated the tumor characteristics of the 67 post-KT UTUC patients based on whether they had native kidney hydronephrosis or not, which can be seen in Table 4. The UTUC in both groups showed a high-grade and muscle infiltrating mode (24/46, 52.2% versus 16/21, 76.2% ≥ pT2). Bilateral UTUC was as high as 41.8% (28/67), but there was no difference between two groups. UTUC subsequent UBUC recurrence was 63.0% (29/46) in the native kidney hydronephrosis group and 57.1% (12/21) in the non-native kidney hydronephrosis group (*p* = 0.569).

## 4. Discussion

Generally speaking, UTUC is relatively rare and accounts for only approximately 2% to 5% of all UC [10]. However, a nationwide analysis of Taiwan has demonstrated unusually high UTUC (>10%) incidence [11]. Unlike Western countries, UTUC is by far the most common form of cancer in post-KT patients in Taiwan [1,2]. Same as in previous studies, our research has revealed that post-KT patients presented with higher incidence of UTUC. Native kidney hydronephrosis and female gender were significantly associated with UTUC. We also observed that the UTUC in our post-KT patients had tendency for bilateral lesions.

Research has suggested that the urine of patients with ESRD contains toxins that may induce carcinoma, especially for UC [12]. Most patients with ESRD with or without KT would have a reduced urine outflow from the native upper urinary tract to the urinary bladder. Consequently, persistent exposure to toxins may occur in the urinary tract. The urinary bladder is located downstream in the urinary tract and has a larger surface area than upper tract. Once patients undergo KT, adequate urine flushes the bladder again. This may partially explain the higher incidence of UC in post-KT patients with more UTUC than UBUC. UTUC after KT often demonstrates an extremely aggressive pathological behavior and frequently goes undiagnosed until an advanced stage. It may result from triggering by immunosuppressive agents. Our cohort also showed about 60% (40/67) of UTUC with stage ≥ pT2 and 83% (56/67) with a high-grade presentation (Table 4). Our post-KT UTUC patients were younger than UTUC patients in the ESRD population and general population (57.6 ± 11.3 versus 62.3 ± 11.6 and 67.6 ± 11.2, respectively) [13,14]. Thus, early detection and subsequent management may help improve oncological outcomes in these patients. Nevertheless, atrophic and native upper tract stricture makes the diagnosis of UTUC challenging and often a biopsy is not harvested due to the difficulty in reaching the lesion of concern. There is low urine efflux from the native upper tract due to ESRD, and urine cytology can therefore mostly detect a urinary bladder lesion. The value of urine cytology in detecting UTUC remains controversial. A washing urine cytology examination still play a role in the standard workup for UTUC; A recent review suggests bilateral upper tract washing cytology is useful to exclude high-grade UTUC in patients with serially indeterminate voiding urine cytology specimens and negative cystoscopies [15].

The clinical presentation of UTUC with kidney hydronephrosis is as usual and easy to understand when kidneys present with continuous urine flush. On the contrary, the consequence between little or no washing effect in native ESRD with UTUC remains unclear. Previous studies have also noted that ESRD patients with UTUC had higher incidence of kidney hydronephrosis [9,16]. There was 68.7% (46/67) post-KT UTUC in patients presenting with either unilateral or bilateral native kidney hydronephrosis in our cohort. Our study further showed the strong association between native kidney hydronephrosis and UTUC in the multivariate analysis (OR, 35.32; 95% CI, 17.99–69.36; *p* < 0.001), but graft kidney hydronephrosis did not. Graft kidney hydronephrosis is mainly caused by surgical complications and most often occurs within the first year after transplantation [17]. The exact mechanism between native kidney hydronephrosis and UTUC needs to be elucidated. We have proposed that the functionally long-term disused upper tract lacks a washing effect, rendering urothelial cells vulnerable to carcinomatous transition and leading to the blockage of the native upper urinary tract. In addition, the post-KT immunocompromised status promotes impaired immunosurveillance, the activation of oncogenic viruses, and has carcinogenic effects [18]. Our data on bilateral kidney hydronephrosis in UTUC and the bilateral UTUC rate are discordant (7/46 versus 21/46) (Table 2 and Table 4). As long as unilateral kidney hydronephrosis prompts obtaining tissue proof of UTUC, subsequent simultaneous bilateral nephroureterectomy and bladder cuff excision are performed. Bilateral UTUC is then confirmed, but the lesion of the latter side is usually too small to cause kidney hydronephrosis on initial presentation. This consideration may explain the discrepancy between the laterality of kidney hydronephrosis and UTUC. Consequently, native kidney hydronephrosis is a predictive factor of UTUC in post-KT patients. Close follow-up and timely intervention are important for post-KT patients with native kidney hydronephrosis.

There are two compound factors in post-KT UTUC: KT and UTUC. Concerning KT, sex disparities on cancer incidence and mortality after KT have been noted and assessed in recent years [19]. It has been described as an up to 20~30% higher risk of cancer for male and white ethnicity transplant recipients [20]. However, several reports comparing gender differences in post-transplantation malignancy rates do not support this data [21,22]. Furthermore, women have been described as having a higher risk and ratio than men for the development of malignancy and KT [1,3,23]. UTUC is a relatively uncommon cancer that accounts for approximately 5% of all UC patients, and in Western countries, men are twice more likely to have UTUC than women [24,25]. However, the female gender has a higher incidence of UTUC in the general population of Taiwan [5,26,27]. In agreement with the two previously mentioned post-KT and UTUC studies, we also found this trend in our cohort (Table 2). Some theories have been proposed to interpret this phenomenon. It has been reported that a higher percentage of compound analgesic and Chinese herb use in female KT recipients may contribute to the higher incidence of de novo UTUC in ESRD patients with or without KT [1,2]. Other factors, such as hormone-related factors and gender differences in the metabolism of carcinogens, have also been raised previously [27,28]. However, hormone receptors, including androgen receptor, estrogen receptor, and progesterone receptor, have been found to be expressed on UTUC cells without gender differences [29,30]. Postmenopausal women have been reported to have less favorable oncological outcomes than men in UC [31]. Another study from Taiwan showed that after adjusting for several risk factors, gender did not appear to be significantly associated with survival outcomes in either UBUC or UTUC [14].

As compared with ESRD without KT patients, post-KT patients with UTUC have more aggressive pathological features and possibly worse oncological outcomes [13,16]. Post-KT patients with native kidney hydronephrosis have clinically presented with persistent hematuria and inconclusive urine cytology yet fail to obtain adequate tissue proof of malignancy. Standard nephroureterectomy and bladder cuff excision should be considered in selected patients. As in previous Chinese and Taiwanese studies [4,32,33], our post-KT patients also had a tendency for bilateral UTUC lesions (41.8%, 28/67) that was much higher than in the general population (2.5%) [34]. Most of these cases were initially diagnosed as unilateral UTUC by URS biopsy or an image study presenting with a unilateral lesion. Coupled with the high contralateral and bladder recurrence characteristics of UC [34,35], we have suggested simultaneous prophylactic bilateral nephroureterectomy and bladder cuff excision in post-KT patients when unilateral UTUC was identified.

The present study had several limitations. First, it was a retrospective cohort study within only one medical center in Taiwan. Incompleteness of urinalysis, cytology, and survival data precluded their further analysis and interpretation. However, we collected more than 1000 cases during our 15-year study period and with a mean follow-up time of 118 months. Second, immunosuppressive agent use is diverse based on different nephrologist care, which makes analysis difficult. Third, native kidney hydronephrosis was only diagnosed by one sonogram or CT scan. We did not trace the kidney hydronephrosis condition during the entire follow-up period. Fourth, not all patient in non-UTUC group had a pathological diagnosis. Forty-two patients with native kidney hydronephrosis were considered to have no UTUC. However, UTUC could occur with longer follow-up period or be too tiny to be identified at that time. Fifth, the causes of ESRD and place of residence in Taiwan of our subjects were unknown. Chinese herbs and analgesic nephropathy may lead to more UTUC. So-called blackfoot disease is an endemic disease caused by the intake of arsenic-contaminated groundwater that has occurred in southern Taiwan. Inhabitants of the blackfoot disease region have presented with much higher UTUC incidence. This may skew our interpretation and analysis. Therefore, we have to speculate about our results using a more conservative explanation.

## 5. Conclusions

Post-KT patients presented with higher UTUC incidence and the onset was younger than in general population of Taiwan and Western countries. Post-KT UTUC is characterized with pathologically aggressive and bilateral lesions in the native kidneys. Native kidney hydronephrosis is strongly associated with UTUC. Failure is common in obtaining tissue proof of native upper UTUC in post-KT patients due to anatomical and technical difficulties. Native kidney hydronephrosis may be a deciding factor for standard nephroureterectomy and bladder cuff excision in selected patients. Nevertheless, almost half of the patients with kidney hydronephrosis do not present with UTUC at the end of our study.

## Figures and Tables

**Table 1:** Characteristics of KT patients.

Characteristic	Total Population *n* = 1005
Transplantation age, y/o	48.2 ± 12.0
Gender	
Male	612 (60.9%)
Female	393 (39.1%)
Smoking	168 (22.7%)
Hypertension	648 (64.5%)
Diabetes mellitus	541 (53.8%)
Dyslipidemia	820 (82.0%)
Hyperuricemia	780 (78.0%)
Hepatitis B virus infection	172 (31.3%)
Hepatitis C virus infection	94 (19.1%)
BK virus infection	281 (39.0%)
Native kidney hydronephrosis	88 (9.4%)
Unilateral	74 (84.1%)
Bilateral	14 (15.9%)
Graft kidney hydronephrosis	217 (23.1%)
UTUC diagnosis, age y/o	57.6 ± 11.3
Average time from KT to UTUC, years	7.35 ± 4.7

KT, kidney transplantation; UTUC, upper urinary tract urothelial carcinoma.

**Table 2:** Characteristics of KT patients with or without UTUC.

Characteristic	With UTUC *n* = 67	Without UTUC *n* = 938	*p* Value
Transplantation age, y/o	51.0 ± 9.6	48.0 ± 12.1	0.046
Gender			<0.001
Male	18 (26.9%)	594 (63.3%)	
Female	49 (73.1%)	344 (36.7%)	
Smoking	7 (11.3%)	161 (23.8%)	0.025
Hypertension	41 (61.2%)	607 (64.7%)	0.561
Diabetes mellitus	41 (61.2%)	500 (53.3%)	0.211
Dyslipidemia	64 (95.5%)	756 (81.0%)	0.003
Hyperuricemia	56 (83.6%)	724 (75.6%)	0.253
Hepatitis B virus infection	15 (32.6%)	157 (31.2%)	0.845
Hepatitis C virus infection	3 (9.1%)	91 (19.8%)	0.130
BK virus infection	19 (39.6%)	262 (38.9%)	0.929
Native kidney hydronephrosis	46 (68.7%)	42 (4.8%)	<0.001
Unilateral	39 (84.8%)	35 (83.3%)	
Bilateral	7 (15.2%)	7 (16.7%)	
Graft kidney hydronephrosis	14 (20.9%)	203 (23.3%)	0.652

KT, kidney transplantation; UTUC, upper urinary tract urothelial carcinoma; KT, kidney transplantation.

**Table 3:** Multivariate logistic regression analysis for the association between UTUC and clinical variables.

Clinical Variable	Coefficient	SE	OR	*p* Value	95% CI	
					Lower	Upper
Native kidney hydronephrosis	3.56	0.34	35.32	<0.001	17.99	69.36
Female	1.21	0.40	3.37	0.002	1.55	7.29
Dyslipidemia	0.91	0.82	2.47	0.272	0.49	12.44
Smoking	0.13	0.54	1.14	0.979	0.40	3.29
Elderly	0.51	0.67	1.66	0.531	0.45	6.17

UTUC, upper urinary tract urothelial carcinoma; SE, standard error; OR, odds ratio; CI, confidence interval.

**Table 4:** Tumor status in KT patients with UTUC.

Status	With Native Kidney Hydronephrosis *n* = 46	Without Native Kidney Hydronephrosis *n* = 21	*p* Value
Histologic grade			
Grade I, Low grade	4 (8.7%)	0 (0%)	0.143
Grade II, III, High grade	36 (78.3%)	20 (95.2%)	0.143
Unknown	6 (13.0%)	1 (4.8%)	
pT stage			
pTis/Ta	5 (10.9%)	2 (9.5%)	0.867
pT1	13 (28.3%)	3 (14.3%)	0.213
pT2	9 (19.6%)	4 (19.0%)	0.96
pT3	15 (32.6%)	12 (57.1%)	0.067
pT4	0 (0%)	0 (0%)	
Unknown	4 (8.7%)	0 (0%)	
Laterality			0.343
Unilateral	25 (54.3%)	14 (66.7%)	
Bilateral	21 (45.7%)	7 (33.3%)	
Recurrence UBUC	29 (63.0%)	12 (57.1%)	0.569

KT, kidney transplantation; UTUC, upper urinary tract urothelial carcinoma; UBUC, urinary bladder urothelial carcinoma.

## Data Availability

The data presented in this study are available on request from the corresponding author with a reasonable reason.

## References

[1] Li W.H., Chen Y.J., Tseng W.C., Lin M.W., Chen T.J., Chu S.Y., Hwang C.Y., Chen C.C., Lee D.D., Chang Y.T. (2012). Malignancies after renal transplantation in Taiwan: A nationwide population-based study. Nephrol. Dial. Transplant..

[2] Wu M.J., Lian J.D., Yang C.R., Cheng C.H., Chen C.H., Lee W.C., Shu K.H., Tang M.J. (2004). High cumulative incidence of urinary tract transitional cell carcinoma after kidney transplantation in Taiwan. Am. J. Kidney Dis..

[3] Chiang Y.J., Yang P.S., Wang H.H., Lin K.J., Liu K.L., Chu S.H., Hsieh C.Y. (2012). Urothelial cancer after renal transplantation: An update. Transplantation Proceedings.

[4] Kao Y.L., Ou Y.C., Yang C.R., Ho H.C., Su C.K., Shu K.H. (2003). Transitional cell carcinoma in renal transplant recipients. World J. Surg..

[5] Yang M.H., Chen K.K., Yen C.C., Wang W.S., Chang Y.H., Huang W.J., Fan F.S., Chiou T.J., Liu J.H., Chen P.M. (2002). Unusually high incidence of upper urinary tract urothelial carcinoma in Taiwan. Urology.

[6] Lin S.H., Luo H.L., Chen Y.T., Cheng Y.T. (2017). Using Hematuria as Detection of Post-kidney Transplantation Upper Urinary Tract Urothelial Carcinoma Is Associated With Delayed Diagnosis of Cancer Occurrence. Transplantation Proceedings.

[7] Kohada Y., Hayashi T., Goto K., Kobatake K., Abdi H., Honda Y., Sentani K., Inoue S., Teishima J., Awai K. (2018). Preoperative risk classification using neutrophil-lymphocyte ratio and hydronephrosis for upper tract urothelial carcinoma. Jpn. J. Clin. Oncol..

[8] Lughezzani G., Burger M., Margulis V., Matin S.F., Novara G., Roupret M., Shariat S.F., Wood C.G., Zigeuner R. (2012). Prognostic factors in upper urinary tract urothelial carcinomas: A comprehensive review of the current literature. Eur. Urol..

[9] Hsiao P.J., Hsieh P.F., Chang C.H., Wu H.C., Yang C.R., Huang C.P. (2016). Higher risk of urothelial carcinoma in the upper urinary tract than in the urinary bladder in hemodialysis patients. Ren. Fail..

[10] Milojevic B., Dzamic Z., Kajmakovic B., Milenkovic Petronic D., Sipetic Grujicic S. (2015). Urothelial carcinoma: Recurrence and risk factors. J. BUON.

[11] Chen J.S., Lu C.L., Huang L.C., Shen C.H., Chen S.C. (2016). Chronic Kidney Disease is Associated With Upper Tract Urothelial Carcinoma: A Nationwide Population-Based Cohort Study in Taiwan. Medicine.

[12] Maisonneuve P., Agodoa L., Gellert R., Stewart J.H., Buccianti G., Lowenfels A.B., Wolf R.A., Jones E., Dsiney A.P.S., Briggs D. (1999). Cancer in patients on dialysis for end-stage renal disease: An international collaborative study. Lancet.

[13] Luo H.L., Chiang P.H., Cheng Y.T., Chen Y.T. (2019). Propensity-Matched Survival Analysis of Upper Urinary Tract Urothelial Carcinomas between End-Stage Renal Disease with and without Kidney Transplantation. Biomed. Res. Int..

[14] Shen C.H., Chiou H.Y., Tung M.C., Wu C.C., Kao W.T., Wang Y.H., Juang G.D. (2017). Clinical and demographic characteristics among patients with urothelial carcinomas of the upper urinary tract and bladder in Taiwan. J. Chin. Med. Assoc..

[15] Zhang M.L., VandenBussche C.J., Hang J.F., Miki Y., McIntire P.J., Peyton S., Vohra P. (2021). A review of urinary cytology in the setting of upper tract urothelial carcinoma. J. Am. Soc. Cytopathol..

[16] Chien C.S., Luo H.L., Ling C.S., Chiang P.H., Chen Y.T., Cheng Y.T. (2016). Upper urinary tract urothelial carcinoma behaviors in patients with end-stage renal disease after kidney transplantation in Taiwan. Int. Urol. Nephrol..

[17] Sui W., Lipsky M.J., Matulay J.T., Robins D.J., Onyeji I.C., James M.B., Theofanides M.C., Wenske S. (2018). Timing and Predictors of Early Urologic and Infectious Complications After Renal Transplant: An Analysis of a New York Statewide Database. Exp. Clin. Transplant..

[18] Halloran P.F. (2004). Immunosuppressive drugs for kidney transplantation. N. Engl. J. Med..

[19] Buxeda A., Redondo-Pachon D., Perez-Saez M.J., Crespo M., Pascual J. (2021). Sex differences in cancer risk and outcomes after kidney transplantation. Transplant. Rev..

[20] Engels E.A., Pfeiffer R.M., Fraumeni J.F., Kasiske B.L., Israni A.K., Snyder J.J., Wolfe R.A., Goodrich N.P., Bayakly A.R., Clarke C.A. (2011). Spectrum of cancer risk among US solid organ transplant recipients. JAMA.

[21] Kim J.H., Kim S.O., Han D.J., Park S.K. (2014). Post-transplant malignancy: A burdensome complication in renal allograft recipients in Korea. Clin. Transplant..

[22] Hsiao F.Y., Hsu W.W. (2014). Epidemiology of post-transplant malignancy in Asian renal transplant recipients: A population-based study. Int. Urol. Nephrol..

[23] Tsai H.L., Chang J.W., Wu T.H., King K.L., Yang L.Y., Chan Y.J., Yang A.H., Chang F.P., Pan C.C., Yang W.C. (2014). Outcomes of kidney transplant tourism and risk factors for de novo urothelial carcinoma. Transplantation.

[24] Lughezzani G., Sun M., Perrotte P., Shariat S.F., Jeldres C., Budaus L., Latour M., Widmer H., Duclos A., Benard F. (2010). Gender-related differences in patients with stage I to III upper tract urothelial carcinoma: Results from the Surveillance, Epidemiology, and End Results database. Urology.

[25] Shariat S.F., Favaretto R.L., Gupta A., Fritsche H.M., Matsumoto K., Kassouf W., Walton T.J., Tritschler S., Baba S., Matsushita K. (2011). Gender differences in radical nephroureterectomy for upper tract urothelial carcinoma. World J. Urol..

[26] Chen C.H., Shun C.T., Huang K.H., Huang C.Y., Yu H.J., Pu Y.S. (2008). Characteristics of female non-muscle-invasive bladder cancer in Taiwan: Association with upper tract urothelial carcinoma and end-stage renal disease. Urology.

[27] Wu Y.T., Luo H.L., Wang H.J., Chen Y.T., Cheng Y.T., Chiang P.H. (2020). Gender effect on the oncologic outcomes of upper urinary tract urothelial carcinoma in Taiwan. Int. Urol. Nephrol..

[28] Zhang Y. (2013). Understanding the gender disparity in bladder cancer risk: The impact of sex hormones and liver on bladder susceptibility to carcinogens. J. Environ. Sci. Health.

[29] Bolenz C., Lotan Y., Ashfaq R., Shariat S.F. (2009). Estrogen and progesterone hormonal receptor expression in urothelial carcinoma of the bladder. Eur. Urol..

[30] Kashiwagi E., Fujita K., Yamaguchi S., Fushimi H., Ide H., Inoue S., Mizushima T., Reis L.O., Sharma R., Netto G.J. (2016). Expression of steroid hormone receptors and its prognostic significance in urothelial carcinoma of the upper urinary tract. Cancer Biol. Ther..

[31] Liu J.Y., Li Y.H., Zhang Z.L., Ye Y.L., Liu Z.W., Yao K., Dong P., Guo S.J., Jiang L.J., Zhong M.Z. (2013). Age-specific effect of gender on upper tract urothelial carcinoma outcomes. Med. Oncol..

[32] Tai H.C., Lai M.K., Wang S.M., Chueh S.C., Yu H.J. (2009). High incidence of urinary tract malignancy among patients with haematuria following kidney transplantation in Taiwan. Transpl. Int..

[33] Liu G.M., Fang Q., Ma H.S., Sun G., Wang X.C. (2013). Distinguishing characteristics of urothelial carcinoma in kidney transplant recipients between China and Western countries. Transplantation Proceedings.

[34] Chromecki T.F., Cha E.K., Fajkovic H., Margulis V., Novara G., Scherr D.S., Lotan Y., Raman J.D., Kassouf W., Bensalah K. (2012). The impact of tumor multifocality on outcomes in patients treated with radical nephroureterectomy. Eur. Urol..

[35] Viart L., Surga N., Collon S., Jaureguy M., Elalouf V., Tillou X. (2013). The high rate of de novo graft carcinomas in renal transplant recipients. Am. J. Nephrol..

